# Exploring parents’ perspectives on feeding their young children: a qualitative study using photo-elicitation in Chile

**DOI:** 10.1017/S1368980022000428

**Published:** 2022-09

**Authors:** Patricia Galvez Espinoza, Marcela Vizcarra, Paulina Molina, María José Coloma, María José Stecher, Kelly Bost, Andiara Schwingel

**Affiliations:** 1 Departamento de Nutrición, Universidad de Chile, Independencia, Chile; 2 Department of Kinesiology and Community Health, University of Illinois at Urbana-Champaign, 1206 S. Fourth Street, Champaign, IL 61820, USA; 3 Centro de Investigación del Comportamiento Alimentario, Escuela de Nutrición y Dietética, Facultad de Farmacia, Universidad de Valparaíso, Valparaíso, Chile; 4 School of Agricultural, Forest and Food Sciences HAFL, Bern University of Applied Sciences, Zollikofen, Switzerland; 5 Junta Nacional de Jardines Infantiles, JUNJI, Santiago, Chile; 6 Department of Human Development and Family Studies, The Family Resiliency Center and Department of Psychology, University of Illinois at Urbana-Champaign, Champaign, IL, USA

**Keywords:** Obesity, Food parenting practices, Preschoolers, Qualitative research, Chile

## Abstract

**Objective:**

Childhood obesity is considered one of the most important public health problems around the world. Chile is currently one of the Latin American countries with a high prevalence of childhood obesity. Given that parents’ food parenting practices shape their children’s lifelong eating habits, addressing those practices is key to curbing later problems of obesity. However, studies of the influences on Chilean parents’ food parenting practices are scarce. Hence, this study explores factors that influence food parenting practices of preschool-aged children in Chile.

**Design:**

Qualitative research, using interviews with the photo-elicitation technique.

**Setting:**

Metropolitan Region, Chile

**Participants:**

Twenty-five parents from families recruited from public childcare centres.

**Results:**

Through a thematic analysis with an inductive approach, we identified five themes that influence food parenting practices: (1) parents’ previous experiences and how they determine their current goals and beliefs; (2) responses to the child’s characteristics; (3) the influences of other family members, especially grandparents; (4) parents’ nutritional knowledge; and (5) living contexts, especially limited budgets and lack of time.

**Conclusions:**

The study reveals multilevel influences, which converge at the family level, on food parenting practices. A family-centrerd approach that addresses the mentioned influences is necessary to improve the management of the childhood obesity problem in Chile.

Childhood obesity is considered one of the most important public health problems around the world^(1)^. By 2016, more than 41 million children under 5 years old were overweight to some degree^(1)^. In Latin America, overweight status (weight-for-height over +2 sd) in children under 5 years old has increased from 6·2 % in 1990 to 7·5 % in 2019^(2)^. These figures are higher than the global prevalence, especially in South America^(2)^. Because of its impact on children’s development and their future as adults^(3)^, new approaches for understanding obesity and its causes are called for, particularly in the early stages of life when healthy eating habits are developed^(4)^.

While Chile’s child undernutrition is the lowest in Latin America, the country has one of the highest rates for overweight in the region (weight for height/length > +2 sd of the WHO Child Growth Standards)^(2)^. Currently, 34 % of Chilean children under the age of 6 years are in the overweight or obese categories^(5)^. Although the goal of reducing obesity in Chile’s preschool population has become a public health priority^(6)^, it has been difficult to achieve. The World Obesity Federation has estimated that despite the implemented government policies such as food marketing regulations for children, Chile has only a 7 % chance of curbing childhood overweight by 2025^(7)^.

Among the causes of childhood obesity, the home food environment is gaining attention^(8,9)^. Family, as part of the home food environment, is described as having a fundamental role in determining whether a child develops obesity^(10)^. Parental influences have been highlighted among family factors because children depend most on them for eating^(4)^, which affects the children’s normal growth and development^(11)^. Parents determine their food parenting practices such as serving sizes, types of foods, and preschoolers’ schedule and frequency of the meals^(12)^, all of which could influence children’s weight status. Beyond this, parents implicitly and explicitly transfer individual and sociocultural meanings to their children (e.g. norms, knowledge, attitudes and behaviours) through interactions with their children during feeding situations. These processes help their children adequately fit into their family, community and culture, while also influencing children’s nutritional status^(13)^.

Food parenting practices vary according to culture, geographic location and socio-economic status (SES)^(14,15)^. For example, Black Afro-Caribbean parents in the UK have been found to use more restrictive food parenting practices than UK White German parents, who use less pressure on their children’s eating practices^(16)^. Studies report that Latino parents from lower SES use more food restrictions and pressure for the child to eat compared to families from higher SES^(17,18)^. Findings reveal several reasons for these differences in food parenting practices. Studies of low-income Latino parents residing in the USA have indicated that they are aware of the importance and are willing to feed their children healthily, but multiple barriers such as tiredness from work, reduced home budgets and time limitations hinder their intentions^(17)^. Other studies have shown that stress can also determine parents’ decisions regarding how to feed their kids^(19,20)^. Although these challenges are not unique to Latino populations, they may be exacerbated by their traditions and culture interacting with their original environment and countries. Despite the importance of food parenting practices and the high prevalence of obesity among the Latin American children’s population, few studies have been conducted in this area. Deepening the understanding of food parenting practices can provide rich information to elucidate this matter in developing countries where childhood obesity rates are rising.

Chilean national data corroborate that young children are eating unhealthily and that there are socio-economic disparities in diet quality^(21)^. Two- to five-year-old children consume low amounts of vegetables and have a high intake of sugar from sugar-sweetened drinks and candies^(21)^. Families from low and middle SES present a lower Healthy Eating Index compared to those from high SES families^(21)^, and preschool children with obesity who attend public early education centres have an excessive energy intake at home during weekends^(22)^. Food parenting practices need to be studied to inform the development of family-centred programmes that more effectively address obesity in young children.

Only one study has explored food parenting practices in preschool children from Chile^(18)^. This study found that children’s BMI was positively correlated with ‘food restriction’ and negatively correlated with ‘pressure to eat’. In-depth knowledge of food parenting practices, their roots and the factors influencing them would contribute to better treatment of childhood obesity in Chile, especially in the low socio-economic population where childhood obesity reaches the highest levels. In this sense, a qualitative approach could enable researchers to address multiple aspects that influence parent–child interactions and understand the topic in a holistic way.

In this context, this study seeks to fill the research gap on food parenting practices in Latin America, particularly in Chile, by learning the reasons why parents feed their young children the way they do. Our goal was to explore the perspectives of Chilean parents from low-income families regarding the food parenting practices they use with their children, using a qualitative approach.

## Methods

### Study design

This research utilises a naturalistic qualitative approach guided by grounded theory elements^(23)^. We selected a qualitative approach because it enabled us to answer questions about ‘the experience, meaning and perspective, most often from the standpoint of the participant’ (p. 499)^(24)^. Our study sought to understand more deeply how parents’ viewpoints on food parenting practices are influenced. Additionally, the foundations of grounded theory allow us to conduct a study without a predetermined hypothesis and develop theories from the data^(25)^. In the current study, we describe the theory from a broad definition that enables researchers to conceptualise, organise, interrelate concepts and give context to explain a phenomenon of interest from the collected data^(26)^. From this theory definition, we present the parent’s perspectives influencing food parenting practices in themes and how they interrelate from the collected data.

The photo-elicitation technique was included because previous research in similar populations has shown that it has some advantages: (a) it offers insights about topics related to food and nutrition^(27)^; (b) it enables the gathering of valuable data that other methods cannot facilitate and (c) it works well in Chilean populations^(28)^.

### Study site and recruitment

This study was conducted in Santiago de Chile, Metropolitan Region. Santiago is the capital of Chile and is the most populated area in the country (more than 40 % of the population lives there)^(29)^.

The researchers had a collaborative relationship with the National Board of Association of Childcare Settings (Junta Nacional de Jardines Infantiles (JUNJI)), a governmental institution that manages childcare settings in Chile. JUNJI provides early education and meal services to children, prioritising those who come from vulnerable families^(30)^; close to 90 % of the children who attend JUNJI childcare centres belong to the two lowest income quintiles^(31)^.

We selected the JUNJI childcare centres that were located in vulnerable neighbourhoods in urban Santiago, according to the Multidimensional Poverty Index^(32)^. For the selected childcare centres (*n* 11), we sent an informative email invitation to principals. This email included a flyer about the study. Nine of the centres agreed to participate. Principals from these centres posted the informative flyer about the study on childcare boards. With the principals’ authorisation, we prepared an invitation letter that briefly explained the research as well as a consent form for the families. Teachers or teacher aides gave this information to parents. In the letter, we asked parents to read the consent form and to indicate whether they wanted to participate; if they wanted to participate, they had to sign the consent and return it to the child’s teacher. After parents returned the signed consent form, we contacted them via phone to check for the inclusion criteria: adult (18 years or older) parents of preschoolers without health conditions that may affect their growth, Chilean nationality, and status as the one who spends the most time with the child outside of school and who interacts with them in food-related contexts (e.g. during dinner). This phone call also allowed parents to ask questions about the research before any activity began.

### Participants

Participants were Chilean parents who spend the most time with the child outside of school and interact with them in food-related contexts (e.g. during dinner). Their children were 3 to 5 years old and had no health conditions that might affect their growth (e.g. food intake affected by cerebral palsy or impaired nutrient absorption). Thirty-two parents were invited to participate, thirty returned the consent form and twenty-five completed all components of the study. Four parents did not finish because of lack of time, and one could not be contacted again.

### Procedures

Semi-structured individual interviews using photo-elicitation were conducted. The research interviewer had two meetings with each parent before the meeting with photo-elicitation. The researcher used the first meeting to collect socio-demographic and anthropometric data, and the second meeting to provide each parent with a disposable camera with a 27-photo capacity. Parents were trained in using the disposable cameras as well as the ethical aspects of taking pictures^(33)^. Then, the researchers asked the parents to take pictures over 7 d, though some took up to 21 consecutive days. Parents received the following instructions: ‘Please take a picture of everything that is important to you regarding how you feed your child, how your child reacts to food and contexts, places, people, or other influences on how your child is being fed’. Telephone calls were used to remind parents about taking photos and answer any questions about the assignment.

The pictures were developed after the collection period. Parents took on average 20 pictures (ranging from 11 to 27 pictures). These pictures were used to guide the final interview. To start, parents were asked to choose seven pictures from their picture set. Then, the SHOWeD technique was used during the in-depth interview^(34)^, which was a method to start the conversation and obtain data that goes beyond what is observable. The technique is an acronym that consisted of the following questions for each selected picture: What do you See in this picture? What was Happening? Does this (problem/situation) happen in Our (your) life? Why does this problem, concern, situation or strength exist? What can we Do about it? Letter ‘e’ ‘does not represent a question’ (p. 26)^(34)^.

During the SHOWeD technique, the parents discussed each of their selected pictures, and the researcher asked questions if more explanation was needed. A questionnaire guide was also developed for the researcher’s use if there was no spontaneous conversation about the photos. In addition to the parents’ selected pictures, the researcher selected five to seven additional pictures to discuss during the interview^(28)^, based on the criteria pertinent to answering the study’s question. The interviewer took field notes during the data collection to catch main ideas from what parents said.

All interviews were conducted in Spanish by a female doctoral student who was trained in the photo-elicitation technique. All procedures were conducted individually in a private place selected by the parents, such as childcare settings or in parents’ homes. All parents gave consent to be audio-recorded during the interviews. Interviews lasted on average 70 min, ranging between 30 and 140 min (only one interview lasted more than 120 min).

### Data analysis

All interviews were transcribed verbatim by native Spanish speakers from Chile, and the leading researcher revised them for accuracy. Field notes taken by the interviewer were used to review the transcriptions and to associate pictures to the participant’s narrative. Data saturation was reached at the 22nd interview because no other topic appeared in the data^(35)^. However, we continued conducting three more interviews to confirm the saturation.

**Fig. 1:**
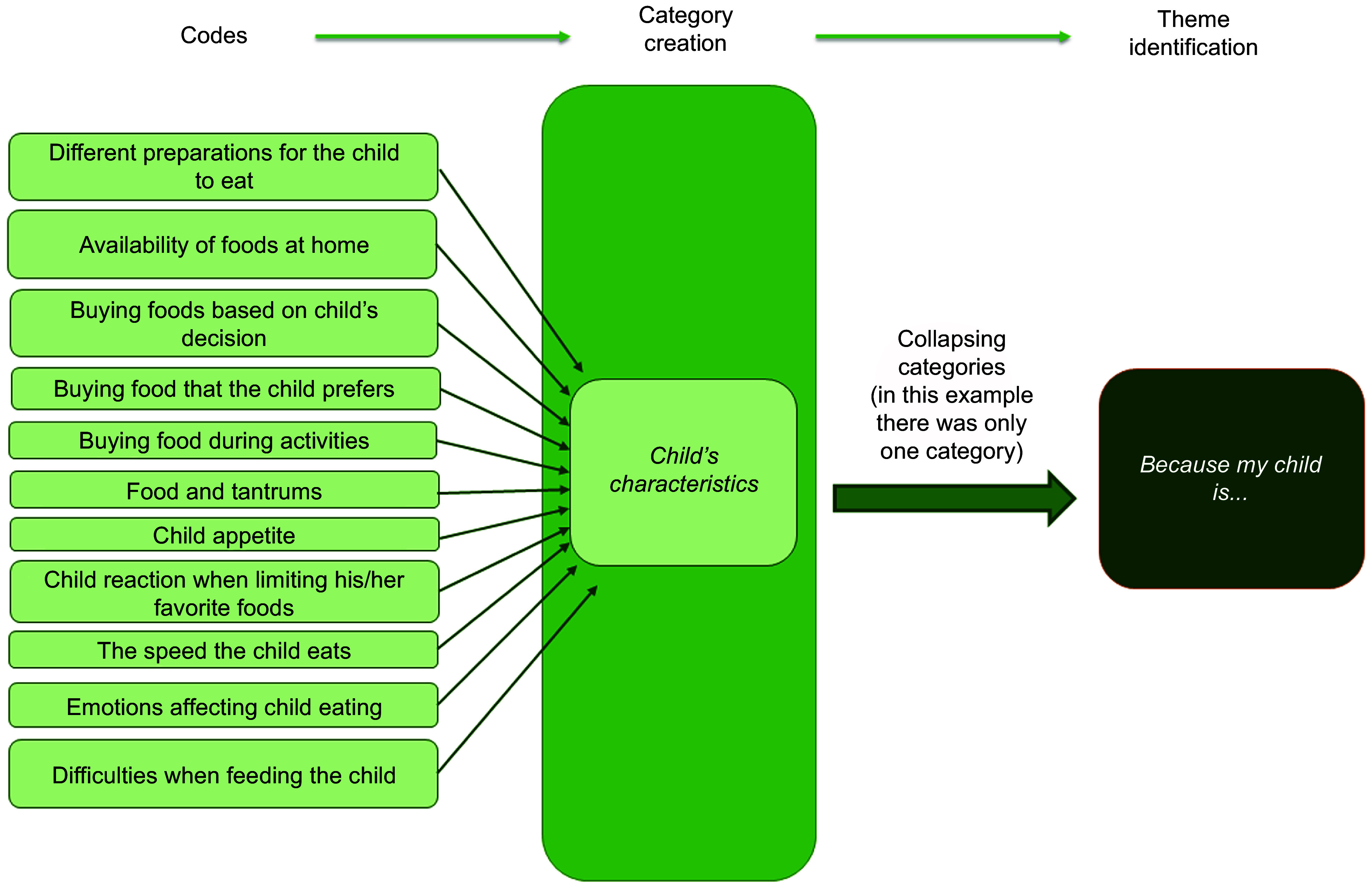
Example of the process of developing the themes

Using an inductive approach, our research team conducted a thematic analysis based on Braun and Clarke’s guidelines^(36)^. Four Chilean nutritionists with experience in the community field and qualitative research conducted a preliminary coding of a subsample of three randomly selected interviews, to familiarise themselves with the data and identify preliminary codes^(37)^. A final codebook was consolidated by the lead researcher after comparing and discussing the preliminary codes, keeping those that were agreed upon by at least three of the researchers^(28)^. The team discussed other codes to evaluate their importance to the research question and to decide whether to include them. All the agreed-upon codes were included in a final codebook; using this codebook, two nutritionists coded each of the interviews. Once all interviews were coded, the team discussed which codes to collapse into categories, according to their similitude or how they were related to each other. Finally, the team reviewed the categories to evaluate which ones to merge or transform into themes for answering the research question^(36)^ (see Fig. 1, as an example of the process of developing the themes). The ATLAS.ti 8, version 8.0.43, was used to organise the data analysis. The involvement of several analysts adds trustworthiness to the study (researcher triangulation)^(38)^. Quotes from the interviews were used as examples of each described theme (See Table 1). All quotes were selected in Spanish, then a bilingual researcher translated them into English. Our research team used the codes to identify quotation origins. Results of the study were shared with parents who could be contacted.

**Table 1:** Summary of each theme and representative quotes

Theme	The most important	Representative quotes
Because my experience says…	Parents’ experiences with their parents and grandparents impacted the repetition of the food parenting practices they found nutritionally adequate and meaningful for their children’s good eating behaviour. Parents also modified those food parenting practices that they did not like as children or what they found inadequate from a nutritional perspective.	I serve small plates for my daughters because my grandmother served me immense servings. I don’t want them to experience the same as me [to eat big dishes]…it was like a dictatorship to eat while I was a child! (NC) I’m not going to be like in the past when we were slapped [to make us eat]. They [her parents] left me eating until 3 or 4 in the afternoon [to finish my meal]. (RB) I was afraid my girl was getting too chubby as I weighed so much in the past. I weighed up to 113kg. (MP)
Because my child is…	Parents’ and children’s characteristics (appetite, temperament, verbal requests, preferences, etc.) and experiences with each other create parent–child feeding dynamics.	[Our child said,] ‘I want to go to the football game with you’ and his dad told him no because it was too dangerous, so he [the child] was angry all afternoon and angrily ate the half of the food and went to his room. (DC) She is very slow to eat, she eats first the dessert, then the salad, and the meal at the end…I’d like her to eat the meal first, but I know it takes her a long time to eat, [so] I let her eat what takes more time to eat at the end. (MG) He [the child] tells me ‘I want oranges…bananas and strawberries’…obviously I buy these fruits…I have bought other fruits, but in the end, I don’t like them, the child doesn’t eat them, the dad eats very little, the fruits get rotten, and we have to throw them away. So for this not to happen, it’s better that we buy what we will eat. (SH)
Because others in my family…	Parents describe how grandparents interfered with their rules related to food and what to expect from grandparents. Parents recognised the help from their parents (the children’s grandparents) when it came to food parenting practices.	When we stay in my mom’s place, it is a battle with her…For example, I don’t put sugar in the child’s milk, but my mom does and then we have discussions…and I tell her ‘the child must drink the milk with no sugar’ and she ‘But, put in a little bit.’ (BS) We don’t get angry [because they give too much food to their grandchildren]. We understand grandparents are meant to spoil their grandchildren; we cannot criticise grandparents because we are going to do the same [when we become grandparents] at certain point. (CT) My mom helped us [as children] to prepare a soup of veggies, mixing in all the veggies we had, and she gave it [the soup] to us, and now I do the same with my child. (DO)
Because I know about it…	Parents have extensive knowledge about nutrients, food components, and healthy foods, and food-related health conditions. The parents pay special attention to childhood obesity and its effects on children, but they also believe some myths.	I learned from fliers in the healthcare centre that if a child does not want to eat, we should not force a child to do it. There are days children eat more and some days they eat less. (DS) My goal when feeding G is that he be healthy and active, [and that] he would not have to eat sugar or things like that, or he [could get] diabetes. (SH) The child needs to drink water to improve his digestion. (PC)
Because I live this way…	Family socio-economic and physical background influence parents’ limited time, and their ability to wisely use the family income for nutritional food selection. Families are also impacted by the availability of technology, their dependence on extended family members, and their reliance on childcare centres to feed their children. All of these factors impact the children’s diet quality.	They give him only healthy food in the childcare centre…they give him beans, [and] carbonada [traditional soup with vegetables, potatoes, and meat]. They give him better meat [than] I can give him at home, because the meat has too much fat, and I don’t like it. (RB) I receive support from the childcare [centre] as I know that in there they give him balanced meals. For me, this is important because I can cook less elaborate meals or I ask the father [of the child] to cook…so I can dedicate time to studying. (JH) I give him some spoons of food, and he [is distracted] with the cartoons [in the TV during meals]…so he begins to eat by himself. (LP)

## Results

Parents’ socio-demographic characteristics are shown in Table 2. The data analysis of parents’ narratives produced five major themes that relate to each other to explain the foundation of the food parenting practices: (1) because my experience says…; (2) because my child is…; (3) because others in my family…; (4) because I know about it… and (5) because I live this way… Parents indicated that their own lived experiences with their families and their particular responses to their child’s eating characteristics were influencing their food parenting practices. The immediate context in which they live, including the family structure and other family member dynamics, influence how parents organise the food environment, creating routines and schedules for the child to eat. These family aspects relate to the limited resources that characterised the parents’ socio-economic background and their families; they depend heavily on their family support to have a place to live and look after their children while the parents work. Finally, parents acquire knowledge from interactions with community organisations, trying to apply it to their family context. Below, a detailed explanation of each theme. A summary of the themes and quotes exemplifying them are summarised in Table 1.

**Table 2:** Parents’ socio-demographic characteristics

Characteristics	Total parents *n* 25	
	*n*	%
Age in years		
20–25	4	16
26–30	8	32
31–35	6	24
36–40	2	8
40–45	3	12
46–50	2	8
Educational attainment		
Elementary	3	12
High school	13	52
Vocational	3	12
Higher education	6	24
Work status		
Unemployed	5	20
Currently working	20	59
Housing		
Own a house	10	40
Rent a house	5	20
Live with other family members	10	40
	Median	Range
Household size	4	2
Type of caregiver		
Mother	24	60
Father	1	4
Marital status		
Two parent family	20	59
One parent family	6	24
Type of health insurance		
Public	8	61
Private	2	8
Both	5	20
	Median	Range
Siblings	1	62

### Because my experience says…

Parents emphasised their life experiences and their beliefs regarding feeding their children. They explained that part of their current food parenting practices had been influenced by past personal experiences (good or bad) while interacting with their parents or grandparents or older siblings. Many parents reproduced the same experiences to feed their children because they thought they were nutritionally adequate and meaningful positive behaviours. For example, one parent indicated

My mom always fed us with vegetables because she always found these foods were cheap in open-air-markets. This is why I think this type of meal [veggies and legumes] made sense [to give to my family]… I also had access to veggies from my granddad. He grew veggies and fruits from the trees that were abundant. (JO)

A few parents said that they tried to prevent their children from experiencing the hunger or economic restrictions that limited access to ‘fancy or better food’ when they were children. The parents tried to give their children everything they could, even if the food was not healthy or if they had to make significant efforts to make it available at home.

Some parents had intentionally modified food parenting practices they did not like that their caretakers had used with them (e.g. forcing a young child to eat all the food served on the plate if s/he is not hungry). A parent mentioned

I always recall I did not like my grandmother’s meals, [and] this is why I do not cook her meals. She cooked a lot of soups…they were like grandmother’s meals. I prefer looking for recipes and I cook them. (NC)

Other parents indicated that they were interested in healthy food parenting practices to prevent the child from becoming as chubby or heavy as they were when they were children. A few parents mentioned the experience of having older siblings who developed health conditions linked to unhealthy eating (e.g. hypercholesterolemia, obesity), which led them to pay more attention to the quality of diet of their younger children.

### Because my child is…

Most parents considered their child’s characteristics when making decisions about food parenting practices. Parents discussed how children’s food preferences, appetite traits, emotional reactions and requests influenced their food parenting practices. All parents were aware of their child’s food likes and dislikes. The child’s food preferences and interests guided the preparation, cooking, and buying of food, the manner of serving food (e.g. order of eating foods within a meal and arrangements of the foods), and the availability of foods at home.

Parents took particular note of their child’s appetite: how much the child usually ate, their selection/rejection of foods and how fast or slow the child ate. Most parents referred to their child as ‘good at eating’ because the child usually ate everything on their plate. Some parents who considered their child’s appetite as good or ‘extremely good’ thought of this as positive, but others used strategies to limit excessive eating by reducing the number of times the child ate unhealthy food. For instance, one parent pointed out

She sits and can eat three yogurts at once or four cookies at once and that’s not normal…I prefer to buy yogurt one by one because the child sees a bunch of it, so she eats a lot. (MO)

They also indicated not being concerned when the child sporadically did not want to eat the whole meal because their child was regularly eating well. However, some parents said that they included foods that the child disliked less frequently and prepared more often those foods that their child enjoyed, particularly when the child was perceived as having regular challenges with eating (e.g. being slow to eat).

Some parents used food parenting practices in response to their child’s emotional reactions, especially to avoid tantrums. To avoid tantrums for not getting foods their child wanted (generally unhealthy and/or expensive food), parents avoided triggering situations, such as places around childcare centres where parents knew they could find some vendors offering the generally unhealthy foods that the child liked. As one parent indicated

When I take him to the childcare centre, he eats two Bonobons [cream covered by a biscuit and chocolate] and it’s not healthy at all. But he has tantrums in the mornings ‘I don’t want to go; I don’t want to go’… (AG).

Additionally, at home, some parents hide foods that children loved without the children noticing. A few parents also negotiated or explained how much of certain foods the child was allowed to eat. In unavoidable situations, such as when other children were eating afterschool, some parents would offer similar or alternative foods that the child wanted (e.g. homemade ice cream).

### Because others in my family…

Parents described multiple ways other family members, especially grandparents, were part of their food parenting practices. Most parents indicated that grandparents directly influenced the interactions that parents have with their children. For example, a parent mentioned

As a mother for the first time…the image of a mother I want to be is my mom. She taught me how to cook the first meals for my child, [and] she has been a tremendous guide [when it comes] to feed[ing] him. (JH)

Parents said that they had some rules for their children about eating, but some grandparents broke the rules and gave the children unhealthy food such as soft drinks, sweets, ice cream, chocolate, cakes, ‘empanadas’ (filled doughs) or fried sweet doughs. Most parents perceived these grandparent behaviours as what grandparents do. However, some indicated that they did not like this demonstration of love and tried to limit the visits from grandparents who were resistant to the parents’ rules. Avoiding grandparents was not a feasible solution for other parents because they lived with them. In consequence, there were conflicts between parents and grandparents concerning food parenting practices. For example, a parent explained

I tell my mom, ‘It’s fine what you are doing, but you have to tell me’…I prohibited something [to eat] to the child, and she gives it to him, so the child is confused. He is already confused, [and] he knows he can get anything from his grandmother. But there is always a limit with me. (PC)

Some parents described grandparents as somebody who guided how they feed their children. Grandparents, in this sense, are seen as a source of knowledge and wisdom. Furthermore, as parents do not have enough time to cook more elaborate food, they saw grandparents as helpful to the diversification of the child’s diet when the grandparents prepared homemade and traditional Chilean meals such as ‘cazuela’ (Chilean soup with whole potato, piece of maize, meat/chicken, vegetables and rice), ‘charquican’ (Chilean beef stew) and ‘pantrucas’ (Chilean soup with a dough made of flour, water, and oil cooked in this soup; veggies and meat can be added).

### Because I know about it…

Most parents described a diverse range of knowledge about food and nutrition that influenced their food parenting practices. This knowledge came mainly from information received from community organisations, such as childcare centres and primary public healthcare centres. A few parents also looked for nutrition information or tried to confirm the information they had received from others in private healthcare centres.

Parents described a variety of foods as healthy, the negative components of foods, the alteration of nutritional properties according to preparation, the relevance of a diverse diet and consideration about amounts of food. As one parent pointed out

I don’t like when kids drink tea; my daughters drink milk or have yogurt. These foods really nourish them; it helps them to grow…I prefer these food options instead of giving them tea. (AG)

Most parents described fruits and vegetables, dairy products, water, and cereals as healthy foods for their children. Additionally, some parents mentioned components of the foods that were inappropriate for their children, such as sugar, colorants, additives and salt. Some parents even made connections between food and health, including diet-related, long-term health conditions such as obesity, hypertension, hypercholesterolemia and diabetes.

One relevant health topic for parents associated with their food parenting practices was childhood obesity. Many parents indicated that obesity was a concern because of its negative impact on health. They mentioned bullying, negative stigma, negative feelings and physical limitations as consequences of childhood obesity. Some parents mentioned encouraging the child to eat healthily to promote healthy growth. Others limited the number of sweets, snacks and other foods to avoid excessive weight gain and obesity-related conditions. However, a few parents said that although their child was overweight, their diet was not a concern; they thought that the child would eventually lose weight at some point of his/her life. One parent explained

I don’t care about that extra one kilogram [in my child] because she sometimes gets sick, generally in the wintertime, and she loses a lot of weight…When they are young children, I’m not that concerned. (MG)

All parents mentioned food labelling, especially food warning labels informing them about critical nutrients. While most parents indicated that these warnings labels were helpful to identify foods with fewer nutrients that affect health negatively, a few indicated that the price of food items was more relevant than food labelling. Critical nutritional characteristics were described by parents when the food warning labels described calories, Na, sugar and fats. Some parents also indicated that foods with a high number of food warning labels were given less frequently and in smaller amounts if they bought or had them at home. A few parents described how their child identified these food warning labels, and this had led the parents to pay attention and to buy foods with fewer food warning labels.

### Because I live this way…

Given limited incomes, parents preferred to buy and prepare foods the children and the family were more likely to eat to avoid wasting food, especially fruits and vegetables. Parents were interested in providing basic meals and foods to feed their families appropriately (usually lunch or bread consumed in a like-dinner meal called ‘once’) instead of special or more expensive food or meals. Special meals were restricted to weekends shared as a family or after receiving payment. Special meals mentioned by parents included pizzas, sushi, hotdogs, hamburgers and fries. For instance, one parent explained

Well…not all Saturdays [we eat hot dogs], when there is money to treat ourselves; otherwise, we eat the bread we normally eat…I prefer buying another [necessary] type of food [rather] than eating so much nonsense. (AS)

Some parents felt a lack of time because of housework, long shifts, and extracurricular activities with their children. This lack of time altered the meal schedule, impacting their preparation of easy-to-make meals and preventing them from arranging or decorating foods to make them attractive to the child. As a result of a lack of time, some parents relied on other family members to feed their child, or they felt less guilty for not giving better quality foods and meals because their child received nutritious meals during childcare hours. One parent indicated

I have always worked late shifts…my brother, my daughter, or the father that arrives at eight at home from work [are in charge of feeding my young child]. So, he [the father] gives [the child] her food and takes care of the rest of things. (MG)

A few parents mentioned strategies to address the challenges with lack of time: for example, one parent cooked meals 1 d ahead, so it was ready when the family needed to eat, while another parent encouraged their child to eat independently.

## Discussion

This study contributes insights into the Latino parents’ perspectives from vulnerable environments when it comes to food parenting practices during the crucial period of parent socialisation of children’s eating behaviours. In this pioneering study in Chile, parents explained their interactions with their children regarding personal, family and other contextual factors in food-related situations. As other studies have reported, families and the children play a key role in food parenting practices, which suggests that from a public health perspective, working with the family as a whole in the domestic environment is necessary to address child feeding behaviours^(39)^. Understanding what influences food parenting practices in Chile and their impact on specific child eating behaviours and weight status may help healthcare practitioners support healthy child growth. This is especially relevant for a country where there are higher levels of childhood obesity rates, and where despite several other efforts, the rates have not improved.

The first influence on food parenting practices was parents’ bad and good childhood experiences that influenced their acquisition of beliefs, goals and meanings related to feeding their children. Two aspects of these past experiences struck our attention. First, parents were not forcing the child to eat everything on their plate. While this feeding practice was frequent in the past, parents now perceived it as unnecessary. This change suggests a cultural shift to adopting an approach responsive to children’s signals related to food. This finding is relevant given that parents can stimulate or interfere with the child’s capability to self-regulate their appetite^(40)^. Parents in our study tended not to force the child to eat and used responsive practices, such as monitoring the child’s communications about hunger and satiety, which is beneficial for their child^(41)^. Second, parents who described suffering from hunger or limited incomes in the past tried to avoid giving the child the same experience, even if this meant not having healthy food at home. Previous studies reported that parents who experienced food insecurity in childhood prioritised their child’s diet over other needs^(42)^ and quantity over quality of food^(43)^. This consequence of food insecurity could be considered a risk factor for childhood obesity because parents may lose control of the amount and quality of foods the children are eating.

Our study also contributes the second influential factor of how children’s characteristics affect food parenting practices. Parents adapted their food parenting practices to their children’s food preferences, appetite or temperament. In line with our results, Webber *et al.* ^(44)^ indicated that in a group of predominantly White mothers residing in the UK, a child’s specific eating behaviours predicted their mother’s food parenting practices. If mothers perceived that their child avoids eating or eats slowly, these children’s behaviours predicted that mothers pressured their children to eat; if children were more food responsive, their mothers had a higher probability of having restrictive food parenting practices. This would mean that parents in our study had a more responsive feeding style, which has been associated with healthier child eating habits^(45)^. This finding needs more investigation because national survey data have demonstrated that children do not have healthy diets^(21)^; it is possible that parents who respond to their kids by providing unhealthy food may partially explain this issue.

A third influence that adds complexity is how other family members affect the feeding parent–child dynamics. Grandparents are described as important influences on parents’ food parenting practices. In our study, many grandparents had a direct influence on feeding the family’s child in a supportive and healthy manner. However, parents were concerned when the grandparents did not promote their grandchildren’s healthy eating. Accordingly, Farrow reported that some grandparents used inadequate food parenting practices, but they also had a substantial repertoire of food practices that benefited the child, which increased their influence according to the time they spent with their grandchild^(46)^. Previous research has also demonstrated that parents and grandparents can have feeding issues between them as a result of their different attitudes, negatively impacting food parenting practices with the child^(47)^. Our study adds knowledge about the positive and negative role of grandparents on food parenting practices and eating behaviours in young children. The grandparents’ role is relevant in Chile and other countries where they have become more involved in taking care of their grandchildren while parents work or need support^(48–50)^. Thus, healthcare providers should consider supporting grandparents to promote a healthy weight in young children because it is a sensitive developmental period for developing food preferences and habits^(50)^. However, to support grandparents, we need to further understand the mechanisms involved in promoting a healthier child weight status through feeding. It is crucial to address the mechanisms that link grandparent feeding behaviours with a children’s risks of being overweight or obese, and those behaviours that are protective.

Parents’ health knowledge was described as a fourth influence on food parenting practices. The relationship of parents’ health or nutritional knowledge and their children’s nutritional health has been widely discussed in the literature^(51,52)^. However, it is relevant to highlight the sources of this knowledge. Parents hold remarkable trust in primary healthcare centres and childcare settings as information sources. In this way, the community environment has a large influence in the home food environment and on food parenting practices. Previous research has shown the role of primary care centres and educational centres in promoting healthy eating in children as well as people of other ages^(53,54)^. This could mean an opportunity and a challenge for healthcare providers and personnel from educational settings in the way they promote healthy eating, because in some cases the providers might be competing with recommendations that parents might find in social media^(55)^.

The dynamics of the parent–child, grandparent–parent and community organisations–parent are more complex when the parents added the context where these interactions occur. Limited income and lack of time were mentioned as influences on the development of a feeding structure. Food budgets determined what they bought and cooked for their children. In addition, a parents mentioned their perception of limited time for cooking, given that they deal with balancing their job and housework; this influences the home food environment because children are more likely to eat less nutritious meals and foods^(56)^. This situation has been described previously in Chilean families from low socio-economic backgrounds^(57)^ and in other parts of the world^(17)^. Public health policies should consider how structural measures, such as reducing prices of healthy food, could reduce barriers for families to eat healthier. Additionally, nutrition policies should consider the support that childcare centres give to families as they deliver food to the children, and how this partially contributes to the socialisation of children’s eating behaviours and to the family food budget.

The current study presents several strengths. First, this research is original in its attempts to thoroughly examine the factors that Chilean parents perceive as influential to their feeding practices during a crucial developmental period for their children. Second, the use of a photo-elicitation interview is a useful method for collecting data in a vulnerable population as it reduces barriers related to education or literacy from participants^(25)^; therefore, it may be applied to similar populations in other contexts.

However, the relevant information gathered by this study has some limitations. It was conducted in a Metropolitan Region of Chile, which may not represent the reality of other regions and rural areas in the country. Additionally, this study’s findings may be applicable to only families with 3- to 5-year-old children who attend childcare centres and reside in low-income neighbourhoods. Finally, we worked mostly with mothers. Further analysis should be conducted to look for distinctions between other caregivers’ perspectives or different kinds of families regarding food parenting practices for infants and adolescents.

## Conclusions and implications

Food parenting practices in this study had multiple influences. We found that parents’ previous experiences, some child characteristics, other family members, nutritional knowledge and the living conditions influenced parents’ decisions about feeding their children. All of the study’s identified themes are interrelated and explain the foundation of the food parenting practices, a vital aspect of the socialisation process of children’s eating behaviours.

As childhood obesity is one of the most critical public health problems in Chile and other parts of the world, policymakers and healthcare providers must use information such as that from the current study to promote young children’s healthy eating. Food parenting practices shape how children eat and are therefore linked to their nutritional status and health. As a result, it may be crucial to guide parents in key decisions regarding these practices, considering the factors that interfere so they can best support children to incorporate healthy eating behaviours from a young age. However, it is necessary to include other family members (beyond mothers) such as grandparents in interventions, taking on the challenges and utilising advantages of a family-centred and culturally competent approach. This approach could be useful for preventing or treating obesity in young children from vulnerable families, especially within Latino cultures in which familism is relevant to health^(58)^. Additionally, public health policies should encourage healthcare centres that use a family and community model to work with childcare settings to promote children’s healthy diet, considering these settings’ valuable role to families. More research is needed to study the best methodologies to work with the family to improve children’s nutritional health.
